# Phenylacetic Acid and Methylphenyl Acetate From the Biocontrol Bacterium *Bacillus mycoides* BM02 Suppress Spore Germination in *Fusarium oxysporum* f. sp. *lycopersici*

**DOI:** 10.3389/fmicb.2020.569263

**Published:** 2020-11-27

**Authors:** Je-Jia Wu, Jenn-Wen Huang, Wen-Ling Deng

**Affiliations:** ^1^Ph.D. Program in Microbial Genomics, National Chung Hsing University and Academia Sinica, Taichung City, Taiwan; ^2^Department of Plant Pathology, National Chung Hsing University, Taichung City, Taiwan

**Keywords:** antagonist, biocontrol, hydroponics, swollen hyphae, volatile organic compounds

## Abstract

*Bacillus mycoides* strain BM02 originally isolated from the tomato rhizosphere was found to have beneficial functions in tomato by promoting plant growth and reducing the severity of *Fusarium* wilt caused by *Fusarium oxysporum* f. sp. *lycopersici* (Fol). Cytological experiments demonstrated *B. mycoides* BM02 reduced Fol invasion by reducing spore attachment and increasing hyphal deformation in hydroponics-grown tomato root tissues. Two volatile antifungal compounds, phenylacetic acid (PAA) and methylphenyl acetate (MPA), were identified from the culture filtrates of *B. mycoides* BM02 by GC-MS analysis. Chemically synthesized PAA, and to a lower extent MPA, suppressed spore germination but have no effects on the hyphal growth of Fol. Our results indicated that the biocontrol agent *B. mycoides* BM02 produced an array of bioactive compounds including PAA and MPA to suppress plant diseases caused by Fol and other pathogenic microorganisms.

## Introduction

Tomato (*Solanum lycopersicum* L.) is an economically important vegetable crop worldwide whose productivity is greatly affected by plant pathogens. Among them, *Fusarium oxysporum* f. sp. *lycopersici* (Fol) infects tomato through root systems and causes discoloration of the vascular tissue, wilting, and eventual death of the plant. Because Fol is a soil-borne fungal pathogen, the common tactics for controlling fungal diseases by fungicides are usually not effective and costly when it applies to the management of *Fusarium* wilt disease. To diminish the use of toxic fungicides with adverse ecological effects on soil microbial communities, biological control employing beneficial microbes could be an effective alternative, and it has been done with some success to manage *Fusarium* wilt disease in tomato (Larkin and Fravel, 1998; Amini and Sidovich, 2010; Barhate et al., 2015). However, due to the complexity in the soil environment and quarantine regulations, it remains a daunting task to apply the bio-products to control *Fusarium* wilt disease in different climate and geographical regions.

Microorganisms produce a wide range of secondary metabolites, some of which are volatile and toxic to plant pathogens. Bacteria produce different volatiles with speculative functions in communication and defense to ensure their survival in various ecological systems (Schulz and Dickschat, 2007). Among them, some are antifungal and have been shown to suppress plant diseases (Wan et al., 2008; Arrebola et al., 2010; Li et al., 2012; Di Francesco et al., 2015; Kanchiswamy et al., 2015). For instance, *Enterobacter cloacae* produced an array of volatile organic compounds, among which ammonia was determined to suppress *Pythium* damping-off (Howell et al., 1988), butyl acetate, phenylethyl alcohol, and 4,5-dimethyl-1-hexene were responsible for reducing the green mold of citrus fruit caused by *Penicillium digitatum* (Chen et al., 2016). Some *Pseudomonas* species produced volatile organic compounds that were toxic to *Sclerotinia sclerotiorum* (Fernando et al., 2005). Volatiles from *Bacillus subtilis* have been shown to induce the formation of abnormal hyphae and inhibit vegetative growth of various fungal pathogens (Fiddaman and Rossall, 1993; Chaurasia et al., 2005; Chen et al., 2008; Chaves-Lopez et al., 2015), which is potentially applicable for managing plant diseases, such as the antifungal volatiles dimethyl disulfide and ammonia from *B. mycoides* capable of preventing cabbage damping-off diseases from *Rhizoctonia solani* and *Pythium aphanidermatum* (Huang et al., 2018).

*Bacillus* spp. are gram-positive, endospore-forming bacteria, which are commonly found in soils and plant rhizosphere. Numerous *Bacillus* strains have been reported to exhibit antimicrobial activities against plant pathogens and effectively reduce diseases on various plants. *B. mycoides* is morphologically unique among all *Bacillus* spp. because it forms a rhizoidal colony with spiral filaments on solid media (Flügge, 1886). As with many *Bacillus* spp., *B. mycoides* is a potential bio-agent by enhancing plant growth and reducing plant diseases due to the elicitation of systemic acquired resistance (Petersen et al., 1995; Bargabus et al., 2004; Czaban et al., 2004a,b). Thus, *B. mycoides* is commonly known as a plant growth-promoting rhizobacterium (PGPR).

In this study, *B. mycoides* BM02 originally isolated from tomato rhizosphere in Taiwan was found to reduce Fol infection in the roots of hydroponic tomato seedlings and subsequently reduced the severity of *Fusarium* wilt disease in the greenhouse. GC-MS analysis identifies two volatile compounds, phenylacetic acid (PAA) and methylphenyl acetate (MPA), from the culture filtrates of *B. mycoides* strain BM02. PAA is a volatile aromatic compound naturally produced by many organisms with antimicrobial, antifungal, and phytotoxic activities. PAA is also endowed with the induction of induced systemic resistance (ISR) (Akram et al., 2016) and hormonal activity similar to IAA *in planta* (Sugawara et al., 2015). Here, PAA and MPA from *B. mycoides* BM02 were demonstrated to be antifungal in suppressing spore germination but not hyphal growth of *Fusarium oxysporum* f. sp. *lycopersici*. Taken together with previously reported dimethyl disulfide and ammonia (Huang et al., 2018), *B. mycoides* may release volatiles as chemical signals in the rhizosphere to promote plant health.

## Materials and Methods

### Microorganisms and Growth Conditions

The BM02 strain of *B. mycoides* Flügge was isolated from the rhizosphere of a tomato plant in Taichung, Taiwan, and has been previously characterized (Peng et al., 2017). Unless otherwise indicated, BM02 was grown on nutrient agar (NA) (per liter contains Beef extract, 3 g; Peptone, 5 g; Agar, 15 g) (BD Difco, Sparks, MD, USA) or in tryptic soy broth (TSB) (per liter contains Pancreatic digest of Casein, 17 g; Papaic digest of Soybean, 3 g; Dextrose, 2.5 g; Sodium Chloride, 5 g; Dipotassium Phosphate, 2.5 g) (BD Difco), and incubated at 30°C. The Fol-04 isolate of *F. oxysporum* f. sp. *lycopersici* (Sacc.) W.C. Snyder and H.N. Hans was recovered as a single spore from the stem of a diseased tomato plant showing wilting symptoms (Lin et al., 2010), and its pathogenicity on tomato was confirmed by Koch's postulates. The internal transcribed spacer (ITS) region of Fol-04 was PCR-amplified using ITS1 and ITS4 specific primers (Toju et al., 2012) and deposited in GenBank under the accession no. MT936807. A Fol-4G isolate constitutively expressing enhanced green fluorescent protein (EGFP) was constructed by introducing p1300-CT74FCir plasmid harboring the *ToxA* promoter-driven *gfp* (Lorang et al., 2001) (kindly provided by Dr. Miin-Huey Lee, Department of Plant Pathology, NCHU, Taichung, Taiwan) into Fol-04 via *Agrobacterium tumefaciens*-mediated transformation as described (Lee and Bostock, 2006). Fungi were routinely grown on potato dextrose agar (PDA) [per liter contains Potato Starch (from the infusion), 4 g; Dextrose, 20 g; Agar, 15 g] (BD Difco) at 28°C. After 1-week plate culturing, fungal spores were suspended in sterile water and filtered through 4-layers of cheesecloth for bioassays.

### Assays for Plant Growth

Seeds of tomato (*Solanum lycopersicum* L.) cv. Farmers 301 were purchased from Known-You Seed (Kaohsiung, Taiwan). Seeds were sterilized by soaking in 70% ethanol for 3 min, followed by 6% sodium hypochlorite (Clorox, CA, USA) for 10 min, washed 5 times with sterile water, and placed on 2% water agar plates. The plates were kept under complete darkness at 25–28°C for 3–4 days. Tomato seedlings were transferred to hydroponic plugs soaking in a Chlorox-disinfected plastic container containing a 700-ml hydroponic solution (Supplementary Table 1). The macronutrients of the hydroponic solution were formulated by Hoagland and Arnon (1938) with some modifications, and the micronutrients were referred to as the basal nutrient solution for *Arabidopsis* as described (Conn et al., 2013). Each plastic container contained three tomato seedlings. For BM02 treatment, the bacterial suspension was adjusted an initial OD_620_ of 1.0 and added into the hydroponic solution at 1,000-fold dilution. All seedlings were grown in a temperature-controlled growth chamber with a daily cycle of 14 h light/28°C and 10 h dark/25°C. Hydroponic solutions supplemented with BM02 bacterial suspension or sterile water were changed once a week to support tomato growth. Seedlings were harvested 4 weeks after the transplant, and the root and shoot fresh weights were measured to record the effects of BM02 on plant growth promotion. Each treatment contained at least 3 replicas, and the experiments were repeated twice.

### Evaluation of Biocontrol Activity

Tomato seedlings with 2–3 true leaves (~2 weeks after transplant) were treated with BM02 by diluting bacterial suspensions at the initial OD_620_ of 1.0 1,000-folds in the hydroponic solution. Seedlings treated with sterile water were used as the mock controls. After 3 days, the treated seedlings were inoculated with Fol-04 or Fol-4G by adding spore suspensions (final conc. 5 × 10^4^ conidia/mL) into the same hydroponic solution. At 4 days post-inoculation (dpi) with Fol spore suspension, the tomato seedlings were transplanted to a 4-inch pot filled with a 1:1 (v/v) mixture of sandy loam and peat moss (BVB Substrate, Bas van Buuren B.V., Coldenhovelaan, The Netherlands), and kept in the greenhouse. The symptoms of tomato *Fusarium* wilt were evaluated for 3 weeks after Fol inoculation using the following scales: 0, no symptom; 1, epinasty; 2, yellowing; 3, wilting; and 4, dead (Supplementary Figure 1). Roots of BM02- or mock-treated tomato seedlings were inoculated with Fol-4G by the abovementioned conditions and collected at 18 h post-inoculation. The root tissues were fixed in 4% paraformaldehyde for 20 min, washed 5 times with phosphate-buffered saline (pH 7.4), and examined by Axio Scope A1 fluorescence microscope (Carl Zeiss AG, Jena, Germany) equipped with a GFP filter (ex/em = 500/535 nm). The autofluorescence of the plant tissues was observed by the Texas Red filter (ex/em = 560/647 nm). Germination of fungal spores and the morphology of hyphae were examined on the 18-mm root segments (*n* > 20) randomly selected from the fixed root samples. Each treatment contained at least three independent tomato seedlings (replicas), and the experiments have been repeated twice. Images were captured as TIF files and analyzed by Carl Zeiss Axio Vision SE64 Rel. 4.9.1 SP1 software (Carl Zeiss AG).

### Purification of Antifungal Compounds From Culture Filtrates

To purify antimicrobial compounds, BM02 was cultured in TSB on a rotary shaker set at 150 rpm at 30°C for 2 days. The pH of culture filtrates was adjusted to 2, 7, and 12, mixed vigorously with ethyl acetate in a separating funnel, and incubated at 25°C for 1 h. Ethyl acetate (EA) fraction was collected and dried at 60°C using a rotary evaporator (Laborota 4000 efficient, Heidolph Instruments, Schwabach, Germany) or Savant SpeedVac vacuum concentrator SC110 (Thermo Fisher Scientific, MA, USA). The EA-extracted, evaporator-dried compounds were dissolved in 100% methanol, passed through a reversed-phase C-18 solid-phase extraction (SPE) cartridge (Bound Elut C-18 column, Agilent Technologies, CA, USA), and eluted using methanol-water solutions as eluents. Methanol was stepwise increased by 10% in each elution. Each eluate was collected, dried, dissolved in 150 μl 100% pure methanol, and tested for antifungal activity.

### High-Performance Liquid Chromatography

The active fraction collected from SPE separation was analyzed further by a model of Agilent 1100 series high-performance liquid chromatography (Agilent Technologies). Compounds were separated in a Thermo Syncronis C-18 column (5 μm, 250 × 4.6 mm, Thermo Fisher Scientific) using a gradient solvent system composed of water-methanol at a flow rate of 1 mL/min as the mobile phase. The concentrations of methanol were programmed as follows: 5–42% at 0–5 min, 42–44% at 5–10 min, 44–45% at 10–25 min, 45–95% at 25–30 min, and hold in 95% at 30–40 min. Compounds were detected by a UV detector with an optical absorbance at 254 nm. Each peak was collected, dried, and tested for antifungal activity.

### Assays for Antifungal Activity

Bioassays for antifungal activity were performed by mixing 1 μl of the abovementioned HPLC fractions dissolved in 100% methanol with 100 μl of Fol-04 spore suspensions (1 × 10^6^ conidia/mL) (1:100, v/v), and placing 20 μl aliquots on a glass slide. Spores treated with 1% methanol only were used as a mock control. Commercially available methyl phenylacetate (MPA) (148442500, Acros Organics, New Jersey, USA) and phenylacetic acid (PAA) (P16621, Sigma-Aldrich, St. Louis, MO, USA) were dissolved in water, serially diluted, and analyzed for their inhibitory activity on Fol-04 by co-culturing with spore suspensions as mentioned and fumigation. Fifty microliters of MPA and PAA at different concentrations were applied to an 8-mm filter paper disc (Advantec Toyo Kaisha, Japan) and placed inside the cap of a Petri dish (15 × 90 mm) to fumigate the spore suspensions on a glass slide. The Petri dishes were sealed with two layers of Parafilm to prevent evaporation. After 12 h incubation at 25°C, both co-culture and fumigation assays were examined for spore germination by a light microscope (Carl Zeiss AG).

### Identification of Volatile Compounds by Gas Chromatography-Mass Spectrophotometry (GC-MS) Analysis

The active fraction (retention time t_R_ = 31.8 min) after HPLC separation was collected, aliquoted, and evaporated to remove the solvent, and subsequently vacuum-dried using SpeedVac (Thermo Fisher Scientific) at 60°C for additional 10, 20, 40, 60, and 90 min. All dried compounds and commercially available MPA and PAA were dissolved in hexane and immediately inserted into the injector (200°C) of a model TRACE GC Ultra gas chromatography (Thermo Fisher Scientific) coupled with a model of ITQ 900 ion trap mass spectrometer (Thermo Fisher Scientific) equipped with an electron capture detector. Helium was used as a gas carrier and flowed at 1 ml/min through a DB-5 capillary column (30 m × 0.25 mm, 0.25 μm, Agilent Technologies). Analytical temperatures were set as follows: initial column temperature at 35°C for 3 min, followed by an increment of 5°C/min up to 200°C and of 20°C/min up to 300°C with a final temperature at 300°C for 5 min. Compounds were further ionized at 70 eV and analyzed by scanning mass spectrometry over a range of 40–600 *m/z* per scan using an ion trap mass spectrometer (ITQ 900, Thermo Fisher Scientific). The identities of volatiles were verified by comparing them with the chemical databases deposited in the GC-mass spectrometry library and by referring the National Institute of Standards and Technology (NIST) mass spectral database using NIST MS Search Program (version 2.0). Quantitation of peak area was conducted by referring to the GC peak area of commercially available MPA and PAA at different concentrations (1, 5, 25, 50, 100, 500, and 1,000 μg/mL) using a linear interpolation method.

### Statistical Analysis

All treatments contained at least three replicas. Data were expressed as means (±SD) and analyzed using IBM SPSS Statistics v 20.0 (IBM, Windows, Armonk, NY). The normality of the variables was examined by the Shapiro-Wilk test. The significance between different treatments was determined by the Mann-Whitney *U* test (*P* < 0.05) for the bioassays on antifungal activities.

## Results

### *Bacillus mycoides* BM02 Reduces *Fusarium* Wilt in Tomato Seedlings

**Figure 1:**
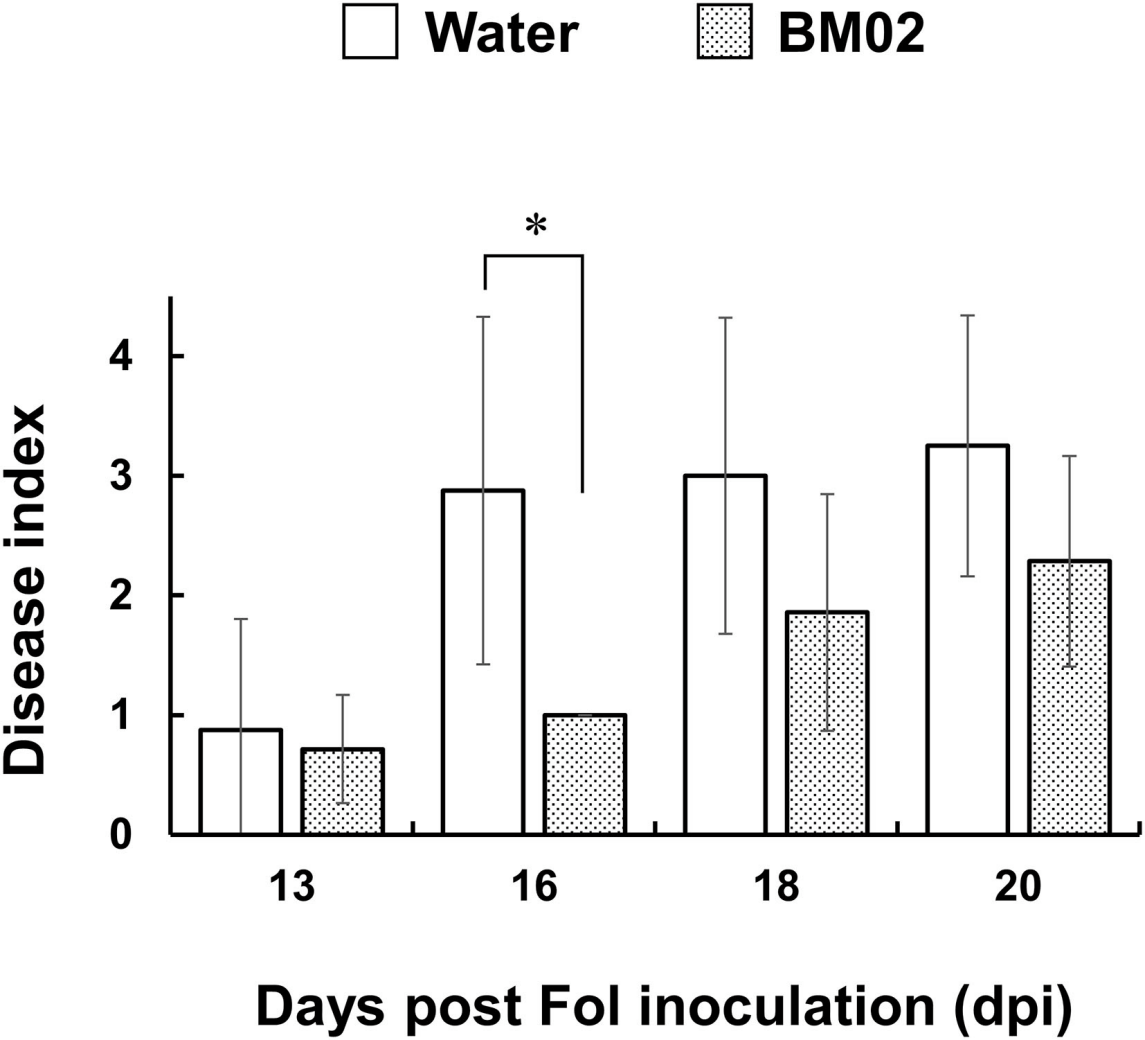
*Bacillus mycoides* BM02 reduces the disease severity of *Fusarium* wilt. Tomato seedlings were grown in a hydroponic system in a disinfected plastic tray. BM02 suspensions (initial OD_620_ = 1.0) were added into the fresh hydroponic solution at the volume ratio of 1:1,000, and the hydroponic solution was refreshed once a week. BM02- or water-treated tomato seedlings were challenged with *F. oxysporum* f. sp. *lycopersici* (Fol-04), disease indices at 13, 16, 18, and 20 days post Fol inoculation (dpi) were shown and compared as indicated. Hydroponic tomato seedlings were inoculated with Fol-04 spore suspensions (final conc. 5 × 10^4^ conidia/mL) 3 days after BM02 treatment, incubated for 4 days in a growth chamber, and transplanted to a 4-inch pot filled with a 1:1 (v/v) mixture of sandy loam and peat moss in a greenhouse. *Fusarium* wilt severity was evaluated daily according to the following rating scales: 0, no symptom; 1, epinasty; 2, yellowing; 3, wilting; and 4, death. Data were the means of seven biological replicas in one representative experiment, and the experiment has been repeated twice to show similar results. Means indicated by asterisks were significantly different according to the Mann-Whitney *U* test (*P* < 0.05).

In hydroponic cultures, tomato seedlings were treated with *B. mycoides* BM02 for 3 days, followed by Fol-04 inoculation for 4 days. The inoculated seedlings were transplanted to a potting mix to monitor the development of *Fusarium* wilt disease in the greenhouse for 3 weeks. In Figure 1, the BM02 treatment led to significantly lower *Fusarium* wilt severity than the non-treated controls (*P* < 0.05), indicating BM02 has beneficial effects on protecting tomato seedlings against Fol infection.

### *B. mycoides* BM02 Exhibits Antifungal Activity Against *F. oxysporum* f. sp. *lycopersici*

**Figure 2:**
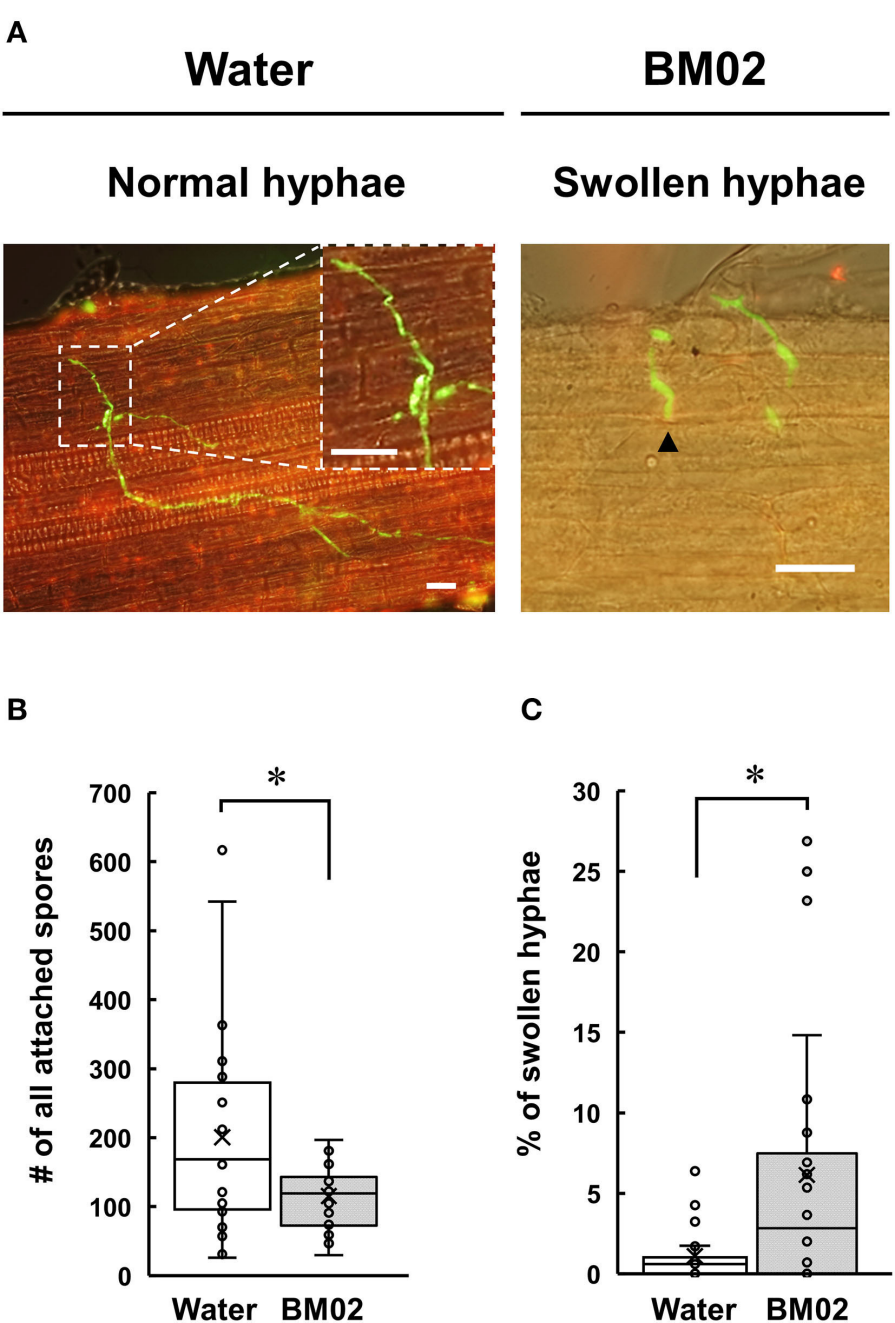
Effects of *Bacillus mycoides* BM02 on spore germination and hyphal growth of *F. oxysporum* f. sp. *lycopersici* (Fol) in tomato. A Fol-4G strain expressing GFP was inoculated to hydroponic tomato seedlings at 3 days after BM02 treatment. Roots were collected and examined by a fluorescence microscope. **(A)** Fol-4G formed swollen hyphae (indicated by black arrowheads in the right panel) on the BM02-treated tomato root. The scale bars in the micrographs are 20 μm. BM02 treatment reduced Fol-4G spore attachment **(B)** and increased the percentage of swollen hyphae **(C)**. Data were the means of 26 (water treatment) and 24 (BM02 treatment) root segments randomly collected from 10 biological replicas in one representative experiment, and the experiment has been repeated twice to show similar results. The percent data in **(C)** were transformed by arcsine square root before statistical analysis using the Mann-Whitney *U* test, and the values indicated by asterisks were significantly different (*P* < 0.05).

Tomato seedling roots inoculated with the GFP-expressing Fol-4G strain were collected and examined by a fluorescence microscope, revealing that the attached spores germinate and produce fungal hyphae with distinct green fluorescence in the root apical tissue (Figure 2A, left panel). The spores attached on the 18-mm root surface were quantified to reveal that a higher number of spores were observed on the water-treated root segments (200.5 ± 144.2 spores per root segment) than the BM02-treated roots (115.4 ± 44.3 spores per root segment) (*P* < 0.01) (Figure 2B). Of the attached Fol-4G spores, their germination in the water- and BM02-treated roots were similar with the percentages of 76.0 ± 20.9 (*n* = 26) and 73.5 ± 15.8 (*n* = 24), indicating the attached Fol spores on the hydroponic tomato root segments were competent for vegetative growth regardless of inoculum density or treatments. Followed by spore germination on the root surface, a small portion of the germ tubes formed swollen hyphae (Figure 2A, right panel). By comparing the fungal structures on tomato root segments upon the treatments of water and BM02, the percentages of swollen hyphae were calculated to be 1.1 ± 1.6 and 6.1 ± 8.2, respectively, which revealed that the BM02 treatment elicited the deformation of fungal hyphae and invasion structures (Figure 2C). The percent data were transformed by arcsine square root to normalize the variance before statistical analysis (*P* < 0.05). By microscopic measurement, the diameter of the swollen hyphae was determined as 2.77 ± 0.52 μm (*n* = 17), much wider than the average of 1.32 ± 0.27 μm (*n* = 81) in the water treatment (*P* < 0.001).

### Purification of Extracellular Metabolites With Antifungal Activity From *B. mycoides* BM02

**Figure 3:**
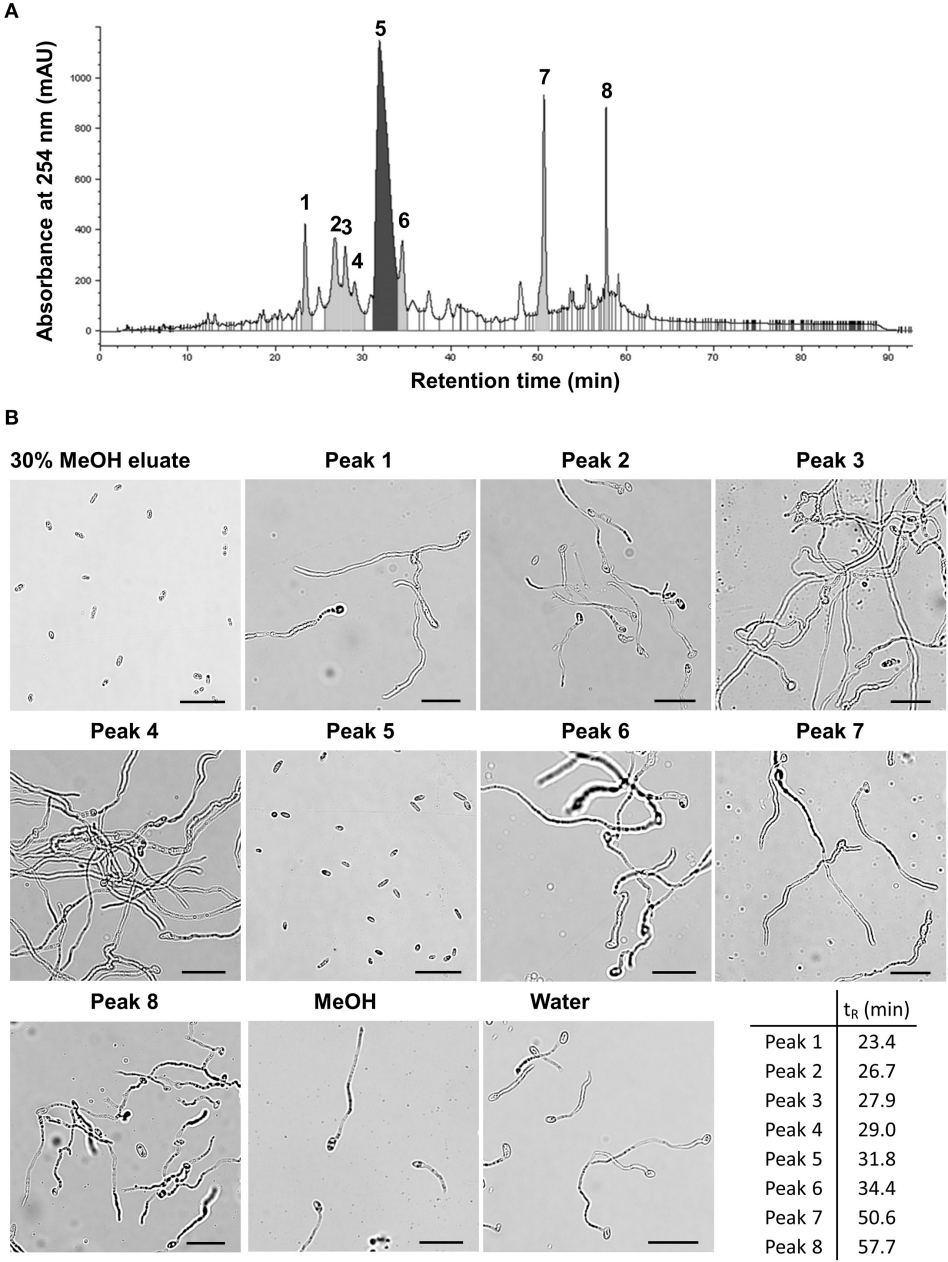
Partial purification of antimicrobial fractions from the culture filtrates of *Bacillus mycoides* BM02 by liquid chromatography. The culture filtrates of BM02 were first extracted by ethyl acetate, and separated by a reversed-phase C-18 solid-phase extraction (SPE) cartridge using water-methanol (MeOH) as the mobile phase, and the fraction of 30% (v/v) MeOH eluate was further purified by HPLC using the procedures in Materials and Methods. **(A)** HPLC chromatogram showed the 30% MeOH eluate of BM02 culture filtrates can be further separated into at least 8 fractions (peak 1-peak 8) by HPLC. **(B)** Bioactivity assays of the 30% MeOH eluate and the HPLC fractions on spore germination in *F. oxysporum* f. sp. *lycopersici* (Fol). Fol spores were mixed with the HPLC fractions at the volume ratio of 1:100 on a glass slide, incubated at 25°C for 12 h, and examined by a light microscope. Spores mixed with water and 1% (v/v) methanol were used as the mock controls. Only the 30% MeOH eluate and the HPLC fraction 5 (peak 5, retention time t_R_ = 31.8 min) completely suppressed Fol spore germination. The scale bars in the micrographs are 25 μm.

BM02 culture filtrates after being grown in tryptic soy broth for 2 days were adjusted to pH of 2, 7, and 12 to produce acidic, neutral, and alkaline crude fractions, and extracted with ethyl acetate (EA). The EA-extracts were tested for the inhibitory effect on spore germination, revealing that Fol spores germinated normally in the EA extracts of neutral and alkaline fractions, but not the acidic fraction (Supplementary Figure 2). The EA extract of the acidic fraction was passed through an SPE C-18 column and eluted using a stepwise methanol gradient (0–100%). Each eluate was collected and tested for the suppression of spore germination, revealing that compounds eluted with 30% methanol effectively suppressed the germination of Fol spores (Supplementary Figure 3). The eluate of 30% methanol elution was analyzed by reversed-phase HPLC, resulting in eight major fractions (Figure 3A and Supplementary Table 2). Bioassays for the suppression of spore germination revealed that only the fraction collected from peak 5 (t_R_ = 31.8 min) had the inhibitory activity on spore germination (Figure 3B).

### Identification of MPA and PAA Produced by *B. mycoides* BM02

**Figure 4:**
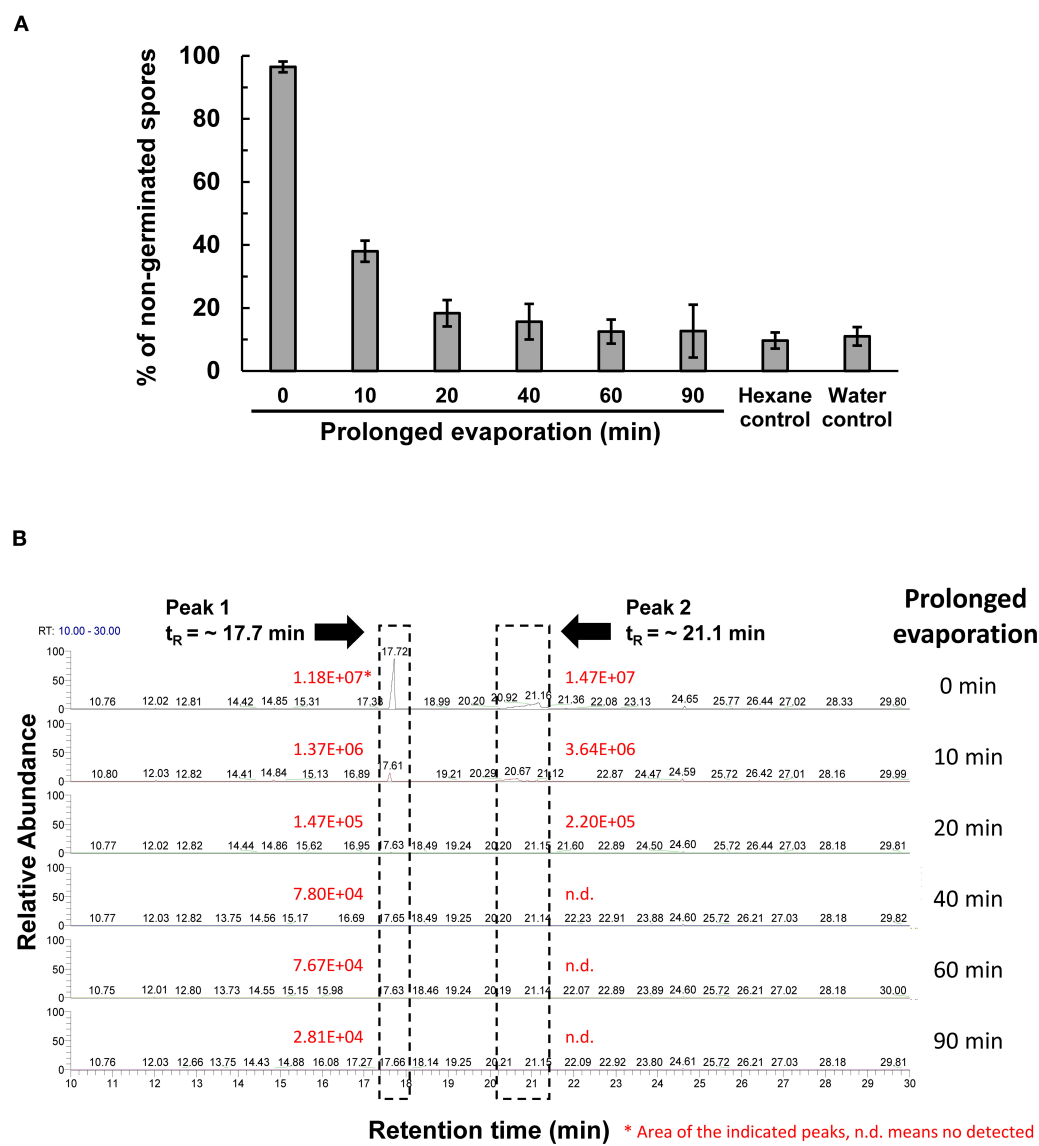
Vacuum evaporation reduced the inhibitory activity of the HPLC fraction 5 on spore germination in *F. oxysporum* f. sp. *lycopersici* (Fol). **(A)** The HPLC fraction peak 5 with inhibitory activity on Fol spore germination shown in Figure 3A was vacuum-evaporated for 0, 10, 20, 40, 60, and 90 min, and the dried solids were dissolved in hexane and immediately tested for the inhibition of Fol spore germination on glass slides. Hexane and water were used as the mock controls. Data were the means of six biological replicas from one representative experiment, and the experiment has been repeated three times. **(B)** Vacuum-evaporated samples were analyzed by gas chromatography. The non-evaporated sample (0 min) yielded 2 peaks at the retention time (t_R_) of 17.7 min and 21.1 min, indicated by dash-line boxes in the GC chromatograms, and the peak areas of the 2 peaks were shown in red. n.d., no detection.

**Figure 5:**
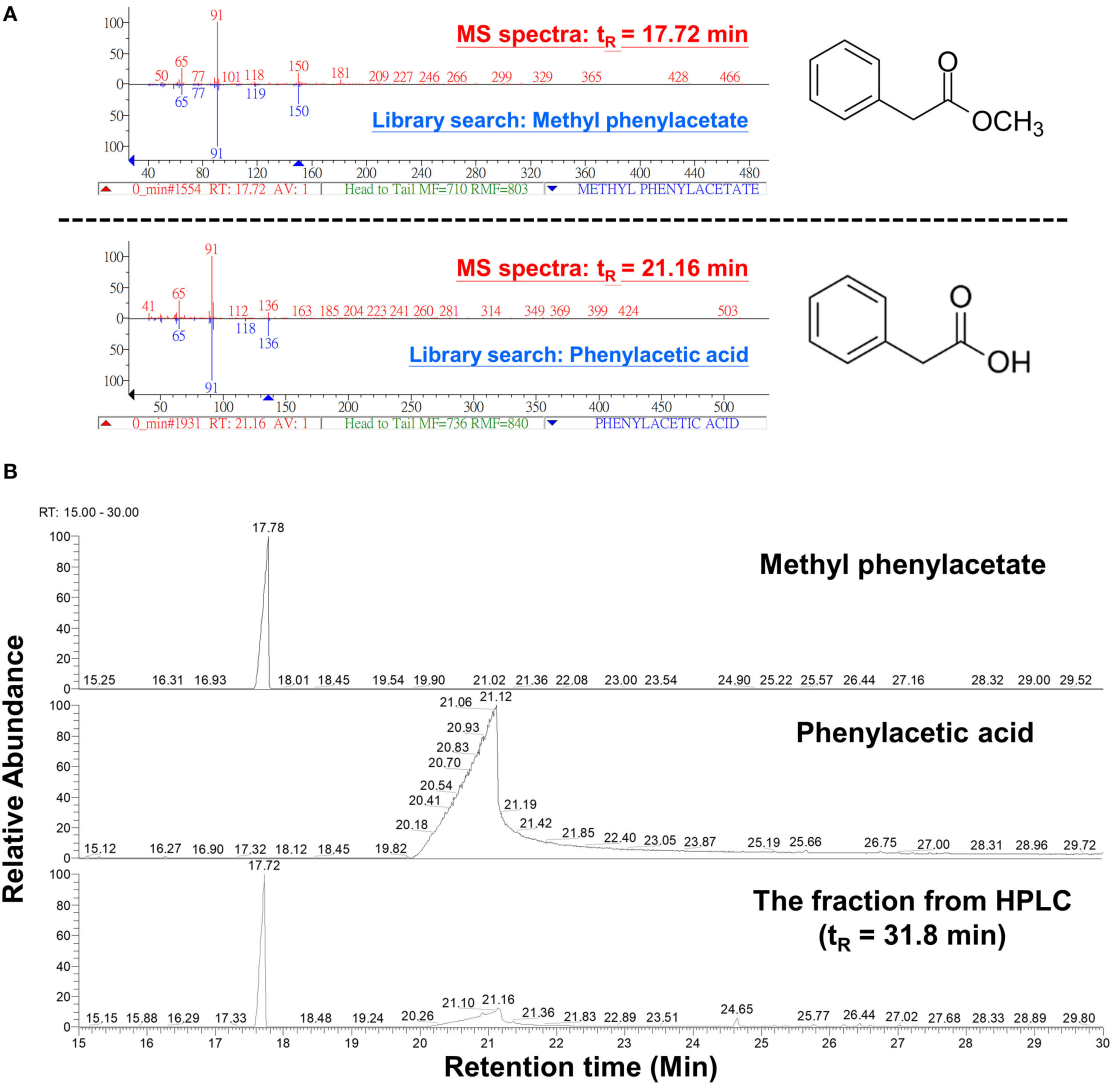
Analysis of volatile compounds in the HPLC fraction 5 by gas chromatography-mass spectrometry (GC-MS) analysis. **(A)** The 2 volatile compounds of t_R_ 17.7 and t_R_ 21.1 min from Figure 4B were analyzed by GC-MS and identified to be methylphenyl acetate (MPA) and phenylacetic acid (PAA), respectively, by comparing with chemical databases deposited in the GC-mass spectrometry library and by reference to the National Institute of Standards and Technology (NIST) mass spectral database using NIST MS Search Program. **(B)** GC chromatograms of the commercial MPA (CAS: 101-41-7, 148442500, Acros Organics), PAA (CAS: 103-82-2, P16621, Sigma-Aldrich), and the HPLC fraction 5 from Figure 3A.

The peak 5 fraction (retention time t_R_ = 31.8 min) collected from HPLC inhibited Fol spore germination and emitted an odor resembling the scent of honey. The inhibitory effect of this fraction on spore germination decreased drastically from 96.5 to 38.0% after being vacuum-evaporated for 10 min and dropped to 18.3% after 20-min evaporation (Figure 4A). GC-MS analysis of the volatile compounds emitted from the peak 5 fraction revealed two major peaks (t_R_ = 17.7 and 21.1 min) (Figure 4B). The area under the peak (peak area, AUC) of these two peaks were found to reduce 100-folds in the first 20 min of heated evaporation. The peak of t_R_ = 21.1 min was heat-labile and not traceable after 40-min evaporation, whereas the peak of t_R_ = 17.7 min showed the AUC of 2.81E+04 after 90-min evaporation (Figure 4B). The peaks of t_R_ = 17.7 and 21.1 min were identified as methylphenyl acetate (MPA) and phenylacetic acid (PAA), respectively (Figure 5A), by reference to the GC-mass spectra database library and further verified by a comparison with the authentic standard and mass spectra of MPA and PAA in the NIST database. The peaks of t_R_ = 17.7 and 21.1 min were detected exclusively from the EA extracts of the acidified (pH 2) culture filtrates of *B. mycoides* BM02 (Supplementary Figure 4). The chemical identities of the two peaks were further validated by comparing to the chemically synthesized MPA and PAA purchased from chemical companies (Figure 5B). The GC peak area of MPA and PAA in different concentrations (Supplementary Table 3) were used as the standards to calculate the concentration of MPA and PAA to be 15.32 and 37.78 μg/mL before evaporation (0 min), which dramatically decreased to 0.94 and 13.87 μg/mL after 10-min evaporation. PAA was no longer detected by GC after 40-min evaporation, however, MPA was still detectable after 90-min evaporation to be 0.02 μg/mL (Supplementary Table 4).

### MPA and PAA Inhibit Spore Germination

**Figure 6:**
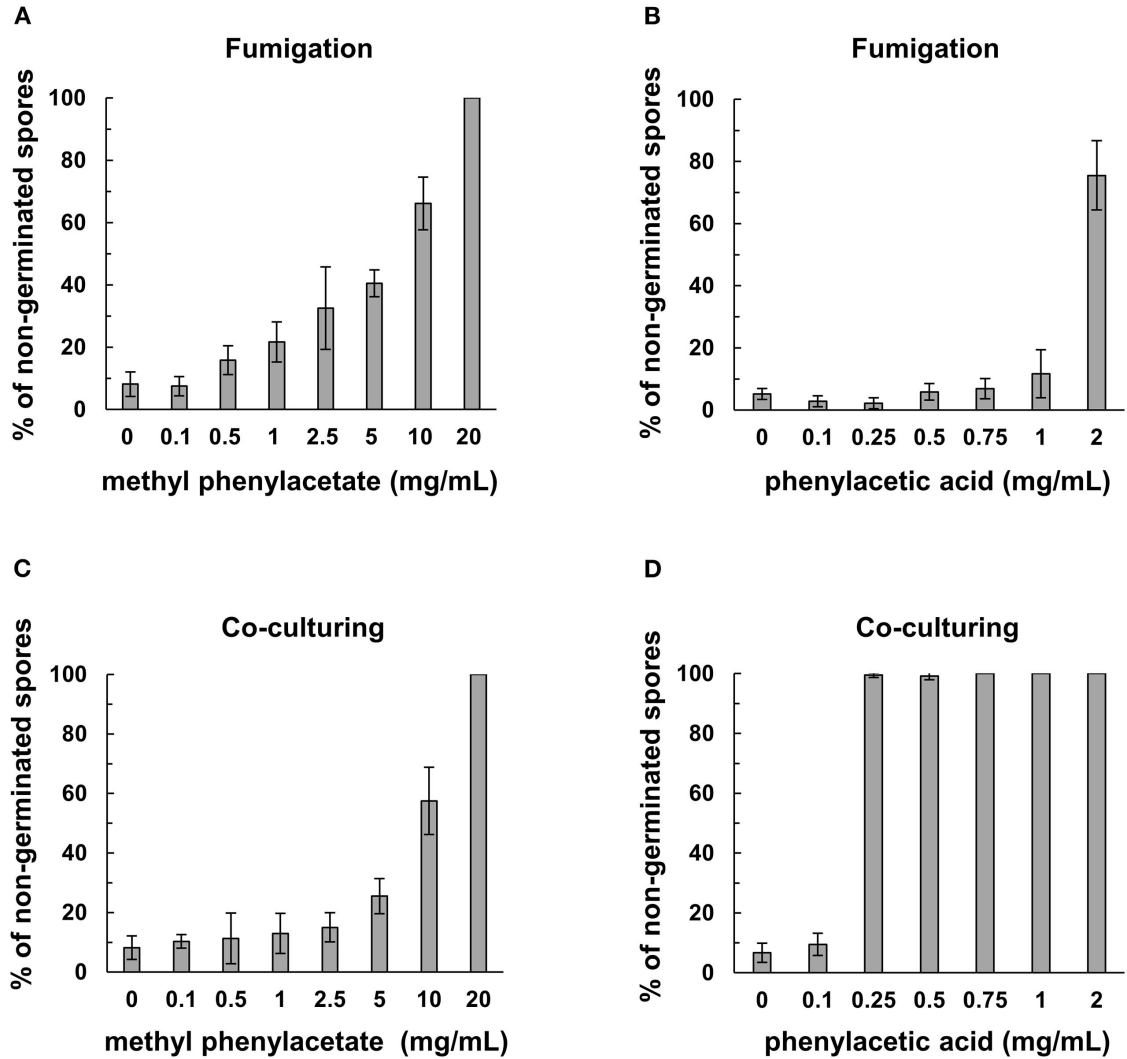
Inhibitory activity of commercial methylphenyl acetate (MPA) and phenylacetic acid (PAA) on spore germination in *F. oxysporum* f. sp. *lycopersici* (Fol). The assays on Fol spore germination were performed in a moist chamber at 25°C for 12 h by fumigation in **(A,B)**, and direct contact in **(C,D)**. Commercial MPA **(A)** and PAA **(B)** were serially diluted and applied on filter paper disks to fumigate the spore suspensions of Fol-04 on a glass slide. For direct contact assays, commercial MPA **(C)** and PAA **(D)** at different concentrations were mixed with Fol-04 spores on glass slides. Spores were observed under a light microscope, and the total number of spores from six randomly selected viewing areas were counted to calculate the proportion (%) of non-germinated spores. Data were the means of 6 biological replicas from one representative experiment.

Co-incubation of the commercial MPA or PAA with Fol spores resulted in a marked inhibition of spore germination (Figure 6). The minimum concentration of MPA to completely inhibit the germination of Fol spores was 20 mg/mL, while 0.25 mg/mL of PAA can reach the same inhibitory activity. Similar assays were conducted by fumigation to show that the concentrations to inhibit 70–80% Fol spore germination were 2 mg/mL for PAA and 10 mg/ml for MPA, indicating PAA is more potent than MPA as an inhibitor to Fol spore germination.

## Discussion

The BM02 strain of *B. mycoides* originally isolated from the tomato rhizosphere was previously shown to produce surfactants and to control *Pythium* damping-off (Peng et al., 2017) and powdery mildew (Dr. Jenn-Wen Huang, unpublished results). Significantly, this study further demonstrated that the BM02 strain protected tomato from Fol infection, and it produced two volatile compounds (MPA and PAA) with antifungal activity. *In-planta* assays showed that BM02 treatment reduced Fol spore attachment and germination, and increased the formation of swollen hyphae, while it did not affect hyphal growth on the surface of the hydroponic tomato roots. GC-MS analysis identified two volatile compounds MPA and PAA that exhibited suppressive effects on the germination of Fol spores. The antifungal activities of MPA and PAA were assayed by co-culture and fumigation on Fol spore suspensions to reveal they were fungistatic (data not shown). Thus, the notion that *B. mycoides* can effectively control plant diseases is likely due to the production of volatile organic compounds and other antimicrobial chemicals, such as surfactants (Peng et al., 2017), ammonia, and dimethyl disulfide (Huang et al., 2018).

PAA has been described to have antifungal and phytotoxic properties. PAA also acted as a plant growth regulator by regulating auxin-responsive genes through the transport inhibitor response 1/auxin-related F-box (TIR1/AFB)–mediated signaling pathway (Sugawara et al., 2015). As an endogenous auxin commonly found in vascular plants and seaweeds (Korasick et al., 2013), PAA promotes the initiation of lateral root primordia in pea seedlings at the optimum concentration of 0.1 mM (Wightman et al., 1980). Moreover, PAA is reportedly produced by a wide range of microorganisms, including *B. licheniformis* (Kim et al., 2004), *B. fortis* (Akram et al., 2016), *Azospillium brasilense* (Somers et al., 2005), *Burkholderia cepacia* (Sopheareth et al., 2013), *Enterobacter cloacae* (Slininger et al., 2004), and *Rhizoctonia solani* (Siddiqui and Shaukat, 2005; Bartz et al., 2013). The microbial production of MPA is less known. Our results showed PAA is more potent as an antifungal chemical than MPA in both the aqueous and gaseous forms. Nevertheless, neither PAA nor MPA has inhibitory effects on hyphal growth. PAA produced by *Streptomyces humidus* has been determined to have a differential inhibitory effect on the vegetative growth of microorganisms, e.g., the growth of *Pythium ultimum* was completely inhibited by 10 μg/ml (~0.074 mM) of PAA, whereas *F. moniliforme* and *F. oxysporum* f. sp. *cucumerinum* were resistant to PAA at the concentration up to 1,000 μg/ml (ca. 7.4 mM) (Hwang et al., 2001). The foliar applications of PAA against Phytophthora blight in pepper plants showed similar protective efficacy as phosphorous acid (H_3_PO_3_), a biostimulant known to suppress diseases and promote growth in horticultural plants, at the concentration of 100–1,000 μg/ml (Hwang et al., 2001). In addition to direct toxicity to the pathogens, root applications of PAA in tomato led to increased quantities of the precursor compounds in the phenylpropanoid pathway, e.g., L-phenylalanine, cinnamic acid, caffeic acid, and salicylic acid, in the shoot tissues, which subsequently triggered induced systemic resistance (ISR) and the biosynthesis of secondary metabolites against *Fusarium* wilt disease (Akram et al., 2016).

*B. mycoides* can produce plant growth regulators and enhance plant growth (Petersen et al., 1995; Czaban et al., 2004a,b; Chen et al., 2010; Ambrosini et al., 2016). Foliar applications of *B. mycoides* and other related species on sugar beet (Bargabus et al., 2002, 2004) and cucurbits (Neher et al., 2009) induced systemic acquired resistance (SAR) and reduced disease severity elicited by fungal pathogens. Root drench of *B. mycoides* revealed that it increased the cell wall thickness of the epidermis and the expression of defense-related genes *PAL* (phenylalanine ammonia-lyase) and *LOX* (lipoxygenase) in tomato roots (Tang et al., 2019), indicating the elicitation of defense responses is a generic trait of *B. mycoides* as a protective agent in crop disease management. Although the chemical elicitors of the defense responses remain elusive, *B. mycoides* produces several volatile compounds including PAA and MPA that may act as the activators of plant defense-related pathways.

After being inoculated onto tomato roots in the hydroponic system, spores of the Fol-4G transformant expressing GFP germinate and grow out on the root surface. Microscopic examination of the fungal structures revealed a spherical ball-like structure was occasionally seen at the tip of the elongating hyphae, and the percentage of the ball-like structure was greatly reduced in the BM02-treated root samples. On the surface of melon radicles infected by a GFP-expressing *F. oxysporum* f. sp. *melonis* transformant, these ball-like structures were formed before the development of infection hyphae in the epidermal cells and named as appressorium-like structures by Inoue et al. (2002). Although the functions of the appressorium-like structures have not been demonstrated in Fol, it is likely to be involved in the early stage of invasion as proposed in other phytopathogenic fungi (Ryder and Talbot, 2015). We also observed Fol hyphae adhered predominantly to the surface of root hairs to initiate the infection cycle, which has been reported previously by inoculating a GFP expressing *F. oxysporum* f. sp. *radicis-lycopersici* transformant to tomato roots in a sterile sand system (Simons et al., 1996; Lagopodi et al., 2002). These results indicated the hydroponic system is useful, particularly for the analysis of tomato-Fol interactions and the elucidation of bio-agents in controlling Fol infection, for its feasibility of microscopic observation of fungal infection structures on well-developed, intact root tissues. Nevertheless, in the later stage of infection, Fol fully colonized the elongation zone of tomato roots in the hydroponics, more extensive than the colonization pattern in soil culture (Olivain et al., 2006; Nahalkova et al., 2008). To evaluate the protective efficacy of BM02 against tomato *Fusarium* wilt, the BM02-treated tomato seedlings in the hydroponics were transplanted to the soil for rating the disease severity under a realistic condition. By comparing with the mock treatment, BM02 greatly reduced the severity of *Fusarium* wilt disease (Figure 1), suggesting the experimental design of combining hydroponics with soil culture is adequate to study plant-microbe interactions in the rhizosphere.

In hydroponic-cultured tomato seedlings, BM02-treated roots had fewer Fol spores adhered on the surface than the water control, but the germination rate remained equal when the spores have successfully attached to the root surface, suggesting BM02 and its metabolites may protect tomato roots from Fol infection by eliciting plant defense response (Tang et al., 2019), as shown by Neher et al. (2009), to reduce spore attachment. In our experimental design, tomato roots were treated with BM02 first, followed by the inoculation of Fol spore suspension. Alternatively, BM02-secreted chemicals in the hydroponic solution may inhibit Fol spore germination prior to root attachment, resulting in the reduction of Fol infection. The swollen hyphae induced by BM02 treatment is a novel structure that has not been observed during Fol infection. Chaurasia et al. (2005) reported *in-vitro* treatment of the diffusible and volatile compounds of *B. subtilis* induced swollen hyphal tips, vacuolization in mycelium, and hyphal lysis of *F. oxysporum* and other fungi; however, similar structures have not been observed on plant tissues. The deformation of fungal hyphae may be induced by several factors, such as cell-wall-degradation enzymes and biosurfactants that elicit pores on fungal hyphae and trigger apoptosis by producing reactive oxygen species (Arlorio et al., 1992; Qi et al., 2010; Oliveira Junior et al., 2012; Vitullo et al., 2012). Likewise, the inhibitors of fungal cell wall biosynthesis could also lead to morphological alteration of fungal hyphae (Grove and Sweigard, 1980; Kang et al., 2001). Alternatively, the chemical compounds produced by BM02, e.g., PAA and MPA, may serve as signals to modulate the defense-related metabolism in root tissues, which retards normal hyphae development and reduces the disease severity of *Fusarium* wilt (Bent and Morton, 1963; Akram et al., 2016).

In conclusion, using a combination of analytical techniques and bioassays, two volatile compounds, PAA and MPA, were identified from the culture broth of *B. mycoides* BM02 that have antifungal activity in suppressing Fol spore germination. Using the combination of the hydroponics and soil culture, BM02 was found to suppress *Fusarium* wilt disease in tomato by reducing spore attachment and increasing the development of swollen hyphae on the root surface, both have profound effects in fungal pathogenesis. Nevertheless, BM02 treatment did not interfere with spore germination *in planta*, suggesting there are other bioactive compounds released by BM02 likely to be involved in the tripartite interactions of *F*. *oxysporum* f. sp. *lycopersici, B. mycoides*, and tomato.

## Data Availability Statement

The raw data supporting the conclusions of this article will be made available by the authors, without undue reservation.

## Author Contributions

The research was designed and directed by J-JW and W-LD. W-LD provided the conceptual and technical guidance. J-JW performed all of the tasks and data analyses. J-WH characterized the biocontrol and plant growth promotion of *B. mycoides*. The manuscript was written by J-JW and W-LD. All authors contributed to the article and approved the submitted version.

## Conflict of Interest

The authors declare that the research was conducted in the absence of any commercial or financial relationships that could be construed as a potential conflict of interest.

## Supplementary Material

Supplementary Figure 1 Disease index of tomato *Fusarium* wilt. Disease severity of tomato *Fusarium* wilt was evaluated by the 5-scale disease indices: 0, no symptom; 1, epinasty; 2, yellowing; 3, wilting; 4, dead.

Supplementary Figure 2 Antifungal activity of the crude extracts from culture filtrates of *Bacillus mycoides* BM02 on Fol-04 spore germination. Culture filtrates of BM02 in TSB were adjusted to pH 2, pH 7, and pH 12, and extracted by ethyl acetate (EA). The EA fractions from different pH were individually collected, dried, and dissolved in 100% methanol for antifungal activity assays by mixing with the spore suspension of Fol-04 at the volume ratio of 1:100. Water was used as a mock control. The mixtures were incubated at 25°C for 12 h and examined under a light microscope. The scale bars in each micrograph are 50 μm.

Supplementary Figure 3 Antifungal activity of C-18 SPE column-fractionated eluates. Acidic culture filtrates of BM02 in TSB (pH 2) were extracted by ethyl acetate and fractionated by reversed-phase liquid chromatography using a C-18 SPE column. Each fraction was individually collected, dried, and dissolved in 100% methanol for antifungal activity assays by mixing with the spore suspension of Fol-04 at the volume ratio of 1:100. For a control experiment, the 1% (v/v) methanol was added into the spore suspension. The mixtures were incubated at 25°C for 12 h and examined under a light microscope. The scale bars in each micrograph are 50 μm.

Supplementary Figure 4 GC-MS analysis of the ethyl acetate-extracted fractions from acidic (pH 2), neutral (pH 7), and alkaline (pH 12) culture filtrates of *Bacillus mycoides* BM02. Each EA fraction was dried and dissolved in 100% hexane for GC-MS analysis. Chemical identification was done by database search using NIST MS Search 2.0.
