# Supplementary material for: Phenylacetic Acid and Methylphenyl Acetate From the Biocontrol Bacterium Bacillus mycoides BM02 Suppress Spore Germination in Fusarium oxysporum f. sp. lycopersici
Source: Front Microbiol. 2020 Nov 27;11:569263. doi: 10.3389/fmicb.2020.569263 (PMC7728801; doi:10.3389/fmicb.2020.569263)
Supplement: Supplementary file 1 [file Table_1.docx]

**SUPPLEMENTARY TABLE 1 | The recipe of Modified Hoagland solution**

| **Macronutrients** | **FW (g/mol)** | **Gm to make**  **1 L stock** | **Stock conc** **(M)** | **Vol of stock**  **(mL) for 1L** | **Final conc** **(mM)** |
| --- | --- | --- | --- | --- | --- |
| NH_4_NO_3_ | 80 | 80 | 1 | **1** | 1 |
| KNO_3_ | 101.1 | 101.1 | 1 | **5** | 5 |
| Ca(NO_3_)_2_•4H_2_O | 236.1 | 118.05 | 0.5 | **5** | 2.5 |
| MgSO_4_•7H_2_O | 246.5 | 123.25 | 0.5 | **4** | 2 |
| KH_2_PO_4_ | 136.1 | 13.61 | 0.1 | **10** | 1 |
| **Micronutrients** | **FW (g/mol)** | **Gm to make**  **1 L stock** | **Stock conc** **(mM)** | **Vol of stock** **(mL) for 1L** | **Final conc** **(**μ**M)** |
| NaFe(III)EDTA | 367.1 | 18.4 | 50 | **1** | 50 |
| H_3_BO_3_ | 61.8 | 3.09 | 50 | **1** | 50 |
| MnSO_4_•H_2_O | 169.02 | 0.85 | 5 | **1** | 5 |
| ZnSO_4_•7H_2_O | 287.5 | 2.875 | 10 | **1** | 10 |
| CuSO_4_•5H_2_O | 249.7 | 0.125 | 0.5 | **1** | 0.5 |
| Na_2_MoO_3_ | 242 | 0.0245 | 0.1 | **1** | 0.1 |
