# Supplementary material for: Phenylacetic Acid and Methylphenyl Acetate From the Biocontrol Bacterium Bacillus mycoides BM02 Suppress Spore Germination in Fusarium oxysporum f. sp. lycopersici
Source: Front Microbiol. 2020 Nov 27;11:569263. doi: 10.3389/fmicb.2020.569263 (PMC7728801; doi:10.3389/fmicb.2020.569263)
Supplement: Supplementary file 2 [file Table_2.docx]

**SUPPLEMENTARY TABLE 2 | The eight fractions by HPLC separation**

| **Peak** | **Retention time**  **(min)** | **Area** | **Height** | **Width** | **Area**  **(%)** |
| --- | --- | --- | --- | --- | --- |
| 1 | 23.4 | 12481.60 | 423.90 | 0.43 | 2.70 |
| 2 | 26.7 | 23890.00 | 370.40 | 0.90 | 5.16 |
| 3 | 27.9 | 15014.80 | 335.50 | 0.62 | 3.24 |
| 4 | 29.0 | 11449.00 | 195.50 | 0.79 | 2.47 |
| 5 | 31.8 | 112452.30 | 1149.00 | 1.22 | 24.30 |
| 6 | 34.4 | 14507.50 | 357.40 | 0.59 | 3.14 |
| 7 | 50.6 | 26445.80 | 933.20 | 0.41 | 5.71 |
| 8 | 57.7 | 11878.40 | 887.00 | 0.18 | 2.57 |
