# Supplementary material for: Phenylacetic Acid and Methylphenyl Acetate From the Biocontrol Bacterium Bacillus mycoides BM02 Suppress Spore Germination in Fusarium oxysporum f. sp. lycopersici
Source: Front Microbiol. 2020 Nov 27;11:569263. doi: 10.3389/fmicb.2020.569263 (PMC7728801; doi:10.3389/fmicb.2020.569263)
Supplement: Supplementary file 3 [file Table_3.docx]

**SUPPLEMENTARY TABLE 3 | The GC peak areas of commercial, serially diluted MPA and PAA**

| **Concentration (μg/mL)** | **Peak Area** | |
| --- | --- | --- |
|  | **MPA** | **PAA** |
| 1 | 1E+06 | ND* |
| 5 | 7E+06 | 8E+05 |
| 25 | 2E+07 | 7E+06 |
| 50 | 4E+07 | 2E+07 |
| 100 | 2E+08 | 1E+08 |
| 500 | 9E+08 | 7E+08 |
| 1000 | 1E+09 | 1E+09 |

*ND, no detection.
