# Supplementary material for: Phenylacetic Acid and Methylphenyl Acetate From the Biocontrol Bacterium Bacillus mycoides BM02 Suppress Spore Germination in Fusarium oxysporum f. sp. lycopersici
Source: Front Microbiol. 2020 Nov 27;11:569263. doi: 10.3389/fmicb.2020.569263 (PMC7728801; doi:10.3389/fmicb.2020.569263)
Supplement: Supplementary file 4 [file Table_4.docx]

| **Prolonged evaporation (min)** | **Compound** | **Apex RT** | **Start RT** | **End RT** | **Area** | **% Area** | **Height** | **% Height** | **Conc.  (μg/mL)** |
| --- | --- | --- | --- | --- | --- | --- | --- | --- | --- |
| 0 | MPA | 17.72 | 17.57 | 17.90 | 1.18E+07 | 44.5 | 2.60E+06 | 88.7 | 15.32 |
|  | PAA | 21.16 | 20.06 | 24.20 | 1.47E+07 | 55.5 | 3.32E+05 | 11.3 | 37.78 |
| 10 | MPA | 17.61 | 17.55 | 17.75 | 1.37E+06 | 27.4 | 4.36E+05 | 73.2 | 0.94 |
|  | PAA | 20.67 | 19.90 | 20.84 | 3.64E+06 | 72.7 | 1.60E+05 | 26.8 | 13.87 |
| 20 | MPA | 17.63 | 17.60 | 17.88 | 1.47E+05 | 40.0 | 4.61E+04 | 62.3 | 0.10 |
|  | PAA | 20.20 | 20.06 | 20.44 | 2.20E+05 | 60.0 | 2.78E+04 | 37.7 | 0.84 |
| 40 | MPA | 17.65 | 17.62 | 17.83 | 7.80E+04 | 100.0 | 2.15E+04 | 100.0 | 0.05 |
|  | PAA | ND | ND | ND | ND | ND | ND | ND | ND |
| 60 | MPA | 17.63 | 17.60 | 17.72 | 7.67E+04 | 100.0 | 2.48E+04 | 100.0 | 0.05 |
|  | PAA | ND* | ND | ND | ND | ND | ND | ND | ND |
| 90 | MPA | 17.66 | 17.63 | 17.77 | 2.81E+04 | 100.0 | 7.15E+03 | 100.0 | 0.02 |
|  | PAA | ND | ND | ND | ND | ND | ND | ND | ND |

**SUPPLEMENTARY TABLE 4 | The peak areas of MPA and PAA in GC chromatograms before and after vacuum evaporation**

*ND, no detection.
