# Supplementary figures and images for: Phenylacetic Acid and Methylphenyl Acetate From the Biocontrol Bacterium Bacillus mycoides BM02 Suppress Spore Germination in Fusarium oxysporum f. sp. lycopersici
Source: Front Microbiol. 2020 Nov 27;11:569263. doi: 10.3389/fmicb.2020.569263 (PMC7728801; doi:10.3389/fmicb.2020.569263)

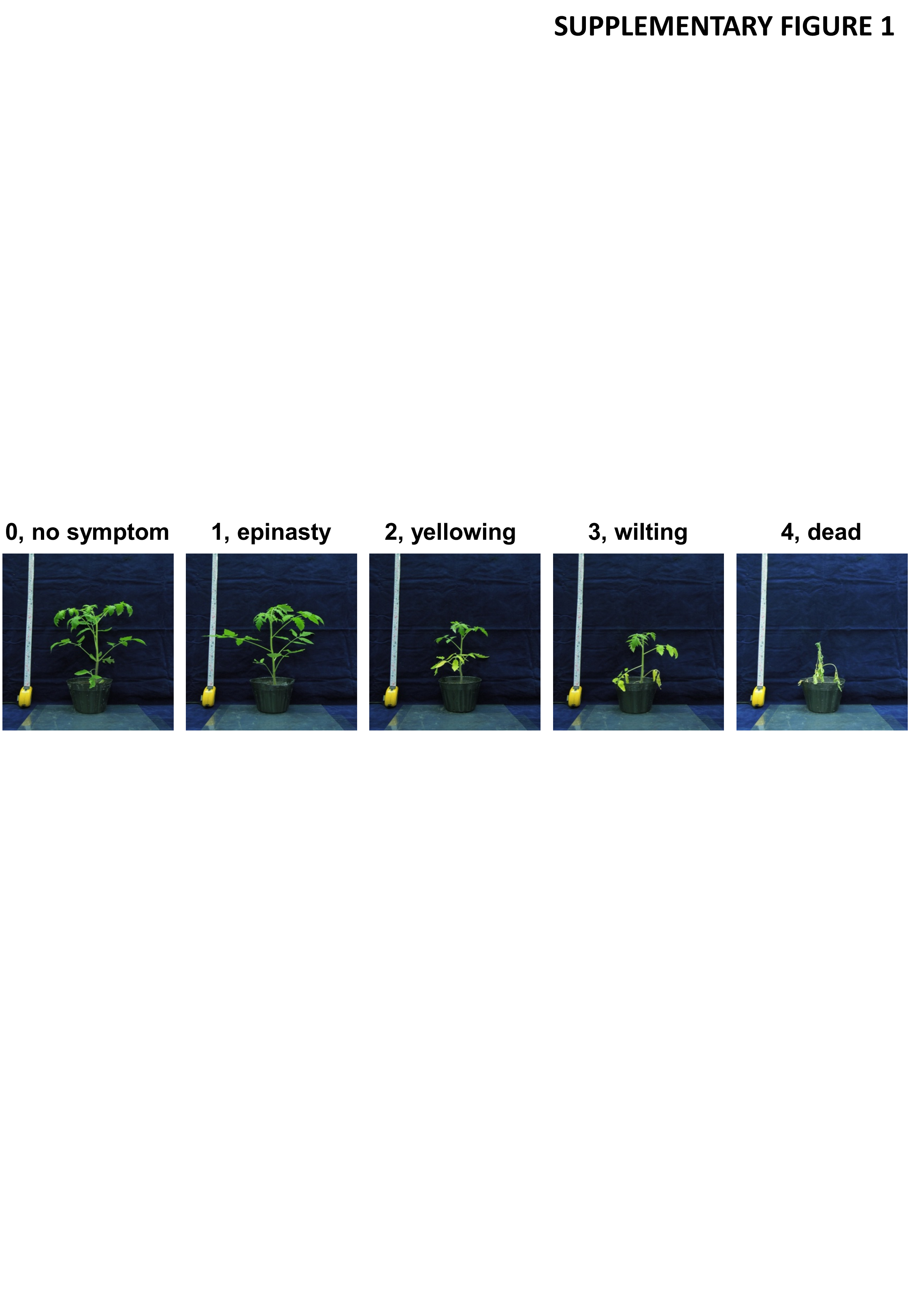

Supplement: Supplementary Figure 1 — Disease index of tomato Fusarium wilt. Disease severity of tomato Fusarium wilt was evaluated by the 5-scale disease indices: 0, no symptom; 1, epinasty; 2, yellowing; 3, wilting; 4, dead. [file Image_1.TIF]

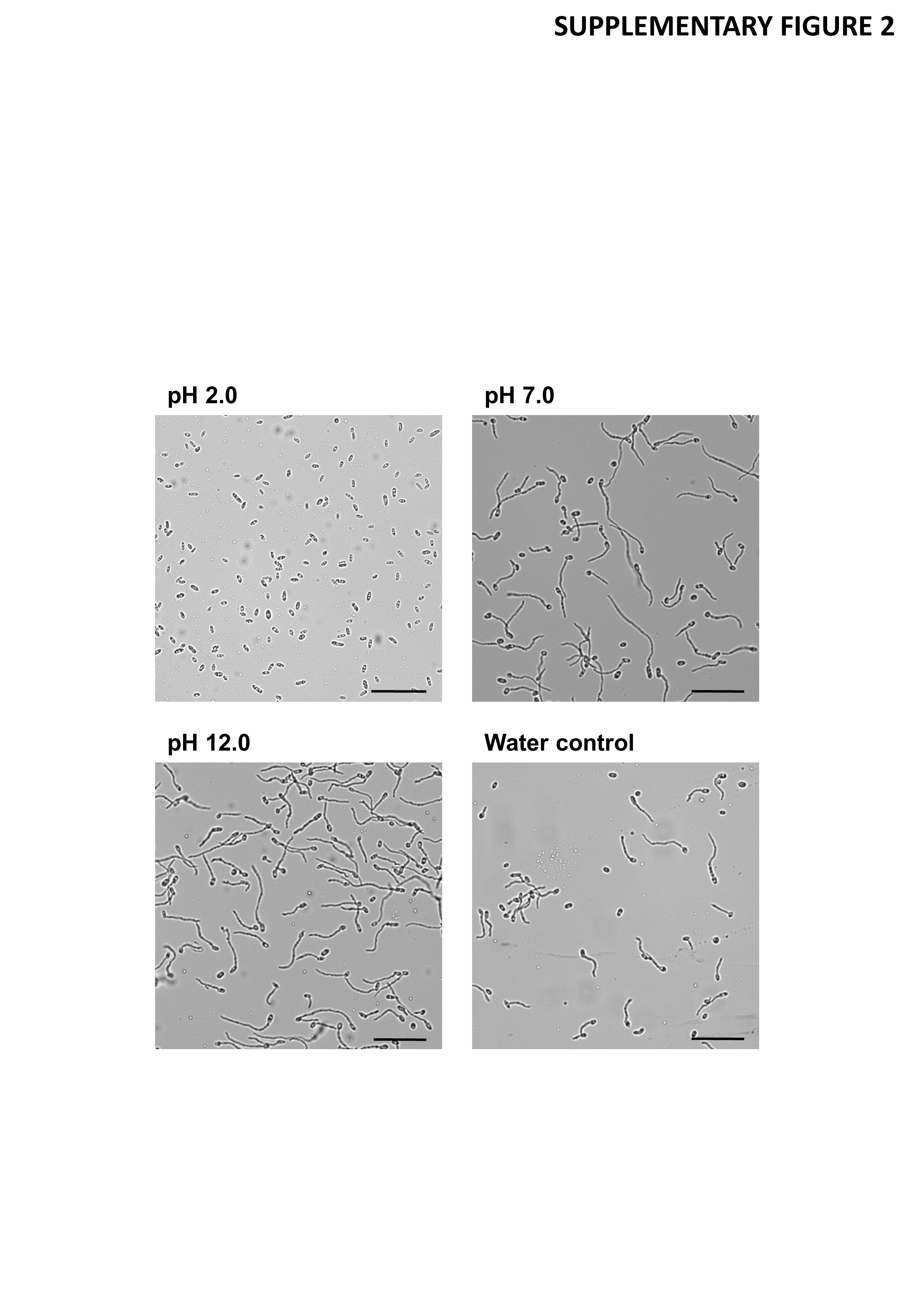

Supplement: Supplementary Figure 2 — Antifungal activity of the crude extracts from culture filtrates of Bacillus mycoides BM02 on Fol-04 spore germination. Culture filtrates of BM02 in TSB were adjusted to pH 2, pH 7, and pH 12, and extracted by ethyl acetate (EA). The EA fractions from different pH were individually collected, dried, and dissolved in 100% methanol for antifungal activity assays by mixing with the spore suspension of Fol-04 at the volume ratio of 1:100. Water was used as a mock control. The mixtures were incubated at 25°C for 12 h and examined under a light microscope. The scale bars in each micrograph are 50 μm. [file Image_2.TIF]

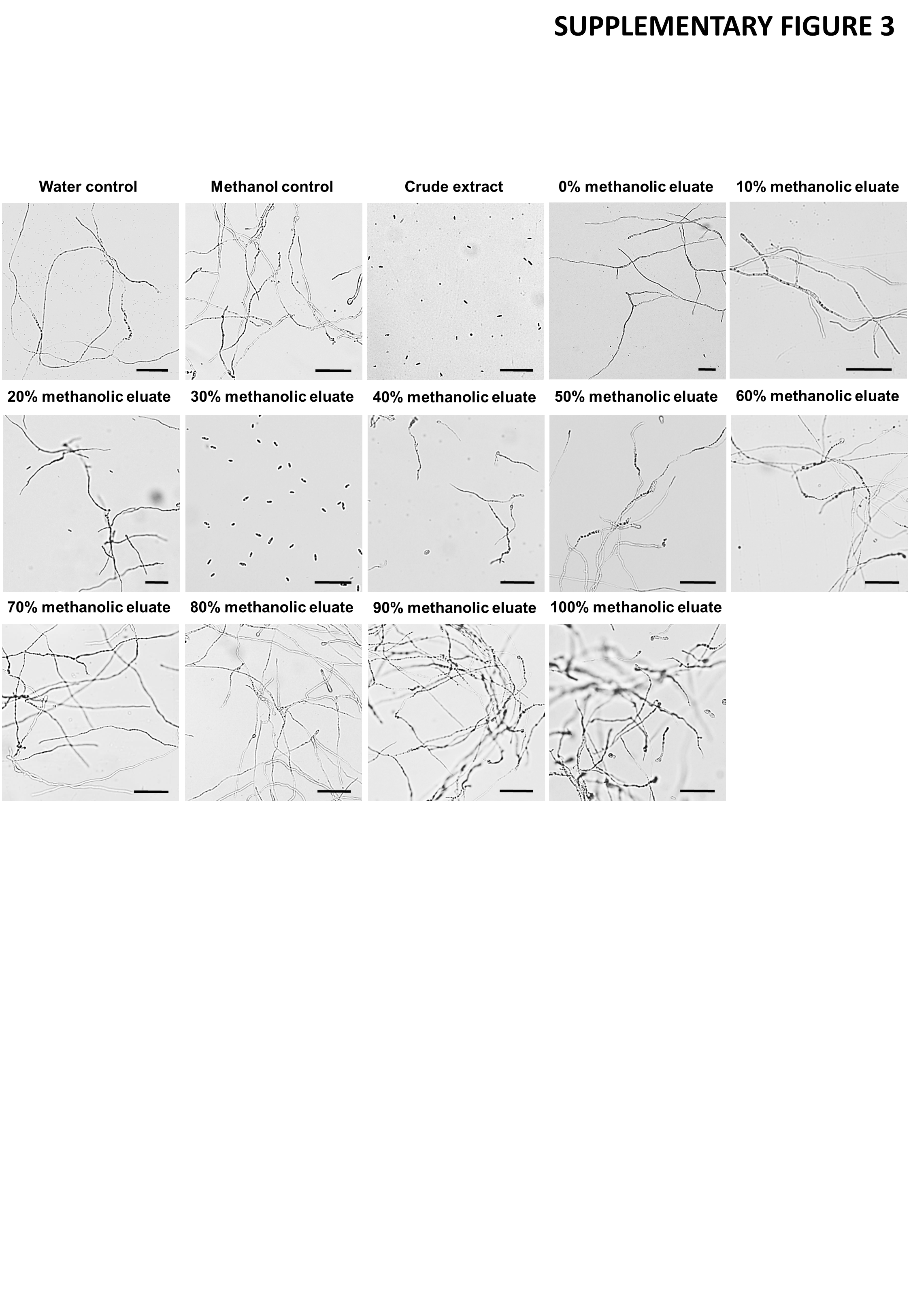

Supplement: Supplementary Figure 3 — Antifungal activity of C-18 SPE column-fractionated eluates. Acidic culture filtrates of BM02 in TSB (pH 2) were extracted by ethyl acetate and fractionated by reversed-phase liquid chromatography using a C-18 SPE column. Each fraction was individually collected, dried, and dissolved in 100% methanol for antifungal activity assays by mixing with the spore suspension of Fol-04 at the volume ratio of 1:100. For a control experiment, the 1% (v/v) methanol was added into the spore suspension. The mixtures were incubated at 25°C for 12 h and examined under a light microscope. The scale bars in each micrograph are 50 μm. [file Image_3.TIF]

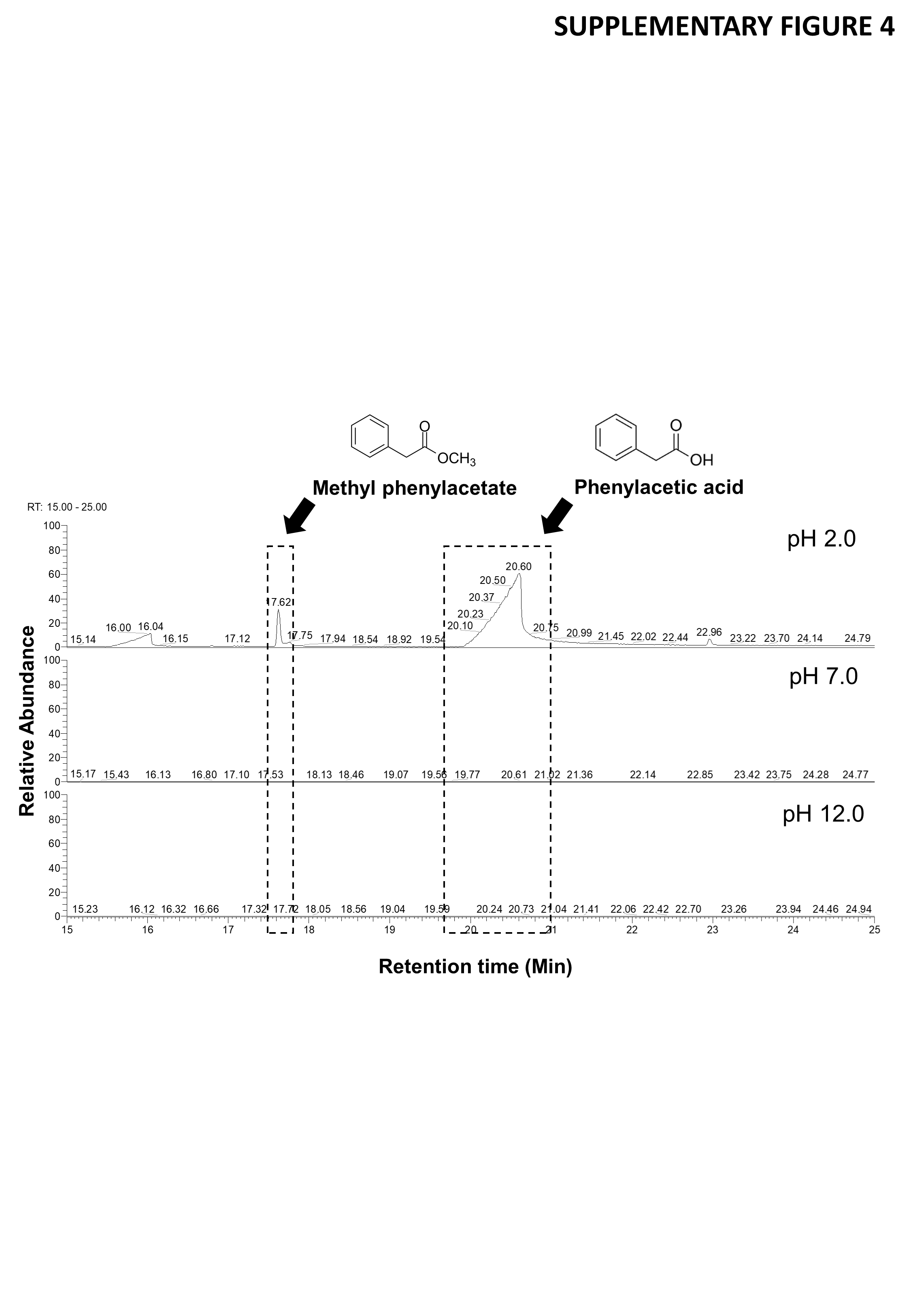

Supplement: Supplementary Figure 4 — GC-MS analysis of the ethyl acetate-extracted fractions from acidic (pH 2), neutral (pH 7), and alkaline (pH 12) culture filtrates of Bacillus mycoides BM02. Each EA fraction was dried and dissolved in 100% hexane for GC-MS analysis. Chemical identification was done by database search using NIST MS Search 2.0. [file Image_4.TIF]
